# eDNA testing reveals surprising findings on fish population dynamics in Thailand

**DOI:** 10.1016/j.heliyon.2023.e17102

**Published:** 2023-06-10

**Authors:** Maslin Osathanunkul, Chatmongkon Suwannapoom

**Affiliations:** a Department of Biology, Chiang Mai University, Chiang Mai, Thailand; b School of Agriculture and Natural Resources, University of Phayao, Muang District, Phayao, Thailand

**Keywords:** Chao phraya river basin, COVID-19 pandemic, Data-poor area, Overfishing, Population decline, Species occurrence and distribution

## Abstract

COVID-19, a global health concern, has an effect on all aspects of the economy. The aquaculture and fishing industries were severely harmed as a result of the closures in multiple nations. Regular systems for inventory monitoring, production, and supply were disrupted. Cancellation of programmes for research, fieldwork, sampling, and tagging influences management-required data. For effective species management, fish dispersion assessments are indispensable. However, due to the difficulty of accessing sampling sites and the associated costs, there is frequently a lack of comprehensive information regarding the distribution and abundance of organisms. The COVID-19 prohibition made fish monitoring more problematic. Due to constant pressure, populations of the stone lapping minnow (*Garra cambodgiensis*), one of Thailand's overfished fish, are rapidly declining. Therefore, eDNA-based monitoring was devised and implemented to reveal the likely dispersal of the species in Thailand prior to and following the lockdown. At 28 locations within the Chao Phraya River Basin, water samples were collected. qPCR was used to determine the presence or absence of *G. cambodgiensis* in water samples. In 78 of 252 water samples, a wide range of computed copy numbers for *G. cambodgiensis* eDNA was observed. It was discovered that samples collected in 2021 (after the lockdown) contain a higher concentration of *G. cambodgiensis* eDNA than samples collected in 2018 or 2019 (prior to the lockdown). The closure appears to be a boon and may result in a substantial restocking of the fish we have studied. Overall, eDNA-based analysis is an extremely promising new survey instrument.

## 1 Introduction

The coronavirus (COVID-19) pandemic is a global health epidemic that is one of the most serious threats to human life. COVID-19 has caused us to alter our behaviour, lifestyle, and beliefs in order to keep it from spreading. Social isolation and remaining at home are required, and previously bustling areas are now deserted. The epidemic has had a devastating influence on all aspects of human existence, including the world economy, which experienced a 3.9% decline in GDP between 2019 and 2020 [1]. The pandemic has had a significant impact on all economic sectors, causing varying degrees of harm across countries and businesses [[2], [3], [4]]. Fishing and aquaculture were also severely affected by the pandemic. Monitoring, stock evaluations, manufacturing, and the supply system were halted, suspended, or disturbed [5]. The harmful effects of COVID-19 on fish population management are unavoidable. According to the [6] study, conservation and management decisions have been significantly impacted. Due to travel limitations, research, field work, sampling, and tagging programmes have been delayed or terminated, affecting both the quality and amount of data required for evaluations. The global disruption caused by the COVID-19 outbreak has impacted inland fishing as many countries go into lockdown. A survey of fisheries experts in 79 countries revealed that the consequences of the pandemic on inland fisheries varied depending on cultural, economic, and public health situations [7]. Several places had reduced fisheries pressure as a result of COVID-19 fallout, such as reduced access to commercial fisheries, fish consumption demand, and tourism. Still, some areas had little change or increased fishing demand [7]. During the COVID-19 pandemic, fish consumption decreased the most compared to other food groups (meat, vegetables, and fruits) [5]. Yet, according to a number of studies, the reduction in demand for fresh fish products in some areas during the pandemic has promoted local exploitation due to a growing desire for fish from local sources [8,9].

The stonelapping minnow (*Garra cambodgiensis*) is a tiny fish of the Cyprinidae family that inhabits Southeast Asian streams and rivers, including the Mekong and Chao Phraya Basins [[10], [11], [12]]. They reside in clean, swiftly moving water with pebbles. The species has a high economic importance for commercial fisheries and is recognised as a regional delicacy. *G. cambodgiensis* is one of the most often consumed freshwater fish in northern Thailand [13]. The taste of the fish, particularly the females with their roes intact, is renowned. A fish can be consumed from head to tail, including its small bones. From June through August, the species migrates to rice fields and floodplains to spawn. During spawning season, the price of fish increases due to increased demand for fish carrying eggs, resulting in a relatively high overall capture of fish. Under continuous pressure from habitat destruction, overexploitation, chemical contaminants, and climate change resulting from the growing human population, *G. cambodgiensis* populations are presently in steep decline [14].

The Department of Fisheries, Ministry of Agriculture and Cooperatives of Thailand has initiated a project for the restoration and protection of rare, endemic, and endangered species in an effort to increase conservation awareness of the imperilled freshwater species. The Fisheries Department has intensified artificial insemination breeding to sustain the species. Over the year of 2018–2020, the Fisheries Department's artificial breeding operation released nearly 4 million individuals of endangered fish species into key water sources in Thailand to increase fish stocks and raise public awareness of the worth of resources. The Fisheries Department has recently (June 2021) selected *G. cambodgiensis* as one of 39 fish to be included in the project for the upcoming fiscal year. The estimation of species distribution and relative abundance is essential for ecological research and environmental assessment [15,16], and thus for measuring the effectiveness of present management efforts. Nevertheless, traditional survey methods frequently necessitate a considerable sampling effort that may cause harm to the target species, are time-consuming, and difficult to estimate on a wide scale [17]. Recently, there has been an increase in interest in monitoring or survey methodologies that use Environmental DNA (eDNA). The eDNA-based method was shown to be a sensitive, effective, and feasible way to detect aquatic organisms, especially rare and elusive species, which in many cases is superior to visual detection or capture method (e.g. [[18], [19], [20], [21], [22], [23], [24], [25], [26]],

Although *G. cambodgiensis* was discovered in the Chao Phraya Basin, no extensive survey or monitoring of the fish in the Basin was conducted. The Chao Phraya Basin is Thailand's largest river basin and one of its most productive river fisheries. The basin is made up of four major tributaries: the Ping, Wang Yom, and Nan rivers, as well as the Chao Phraya River. Here, the eDNA-based approach was used to determine the presence of *G. cambodgiensis* in all five rivers of the Chao Phraya Basin (Ping, Wang, Yom, Nan, and Chao Phraya Rivers). The eDNA surveys were conducted both during and after the COVID-19 pandemic lockdown. The findings would be important for assessing the impact of the pandemic on the species, identifying and capitalising on possibilities to improve local fisheries management, and identifying key habitats and developing a fish conservation programme if necessary.

## 2 Materials and methods

### 2.1 Water sampling and DNA extraction

**Fig. 1:**
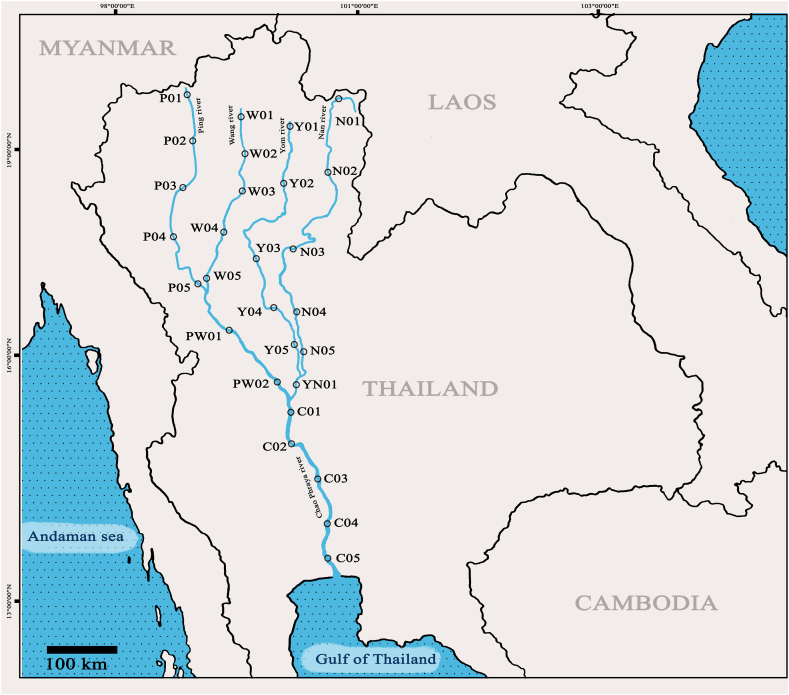
Map of 28 sampling sites located on Chao Phraya River Basin.

Waters samples were collected from the surface and near the river centre at 28 sites in the Chao Phraya Basin in triplicates at each site (Fig. 1 and Table 1). Sample collections were conducted in November–December 2018, 2019, and 2021. The sampling did not carry out in year 2020 due to COVID-19's travel restriction. Water sampling and DNA extraction were conducted accordingly to Ref. [27]. In order to prevent contamination, all field equipment was sanitised using 10% bleach (0.6% sodium hypochlorite), UV-Crosslinker, or an autoclave and sealed. For each sample, a different pair of nitrile disposable gloves was utilised. At each location, water samples were collected in a container that had been decontaminated with a 10% bleach rinse and two distilled water rinses. Water samples of 100 mL were quickly filtered in the field using a BD Luer-LokTM syringe (sterilized with UV light for 1 h before use and individually sealed) equipped with a glass fibre 0.7 m filter (Whatman GF/F); nine samples per replication were taken at each site (n = 28 sites x 3 replications x 9 filters = 756 filters for each sampling event year). In the same way that water samples were filtered, 100 mL of distilled water was filtered for each site (nine filters) as a field-negative filtering control. Filters were then placed in 1.5 mL microcentrifuge tubes with tweezers and maintained in a polystyrene box containing dry ice before being transferred to a 20 °C freezer until extraction. To extract one eDNA sample, nine filters were combined. All samples were extracted within 48 h of collection in a dedicated clean laboratory following the DNeasy Blood and Tissue Kit (Qiagen, Hilden, Germany) method from Ref. [27]. Samples were eluted twice in 25 μL of TE buffer and then stored at 20 °C. To eliminate potential PCR inhibitors, each sample was processed with the OneStep PCR Inhibitor Removal Kit (Zymo Research).

**Table 1:** Sampling site details.

ID	River	Coordinates	Elevation (m)	Proximity to major urban areas (m)
**P01**	**Ping**	19.586484, 98.936944	475	150–200
**P02**		19.072405, 98.941783	329	200–300
**P03**		18.423255, 98.698165	273	100–150
**P04**		18.092970, 98.593880	251	1000
**P05**		17.238374, 99.026320	141	600–700
**PW01**		16.453455, 99.520941	72	0
**PW02**		15.850660, 100.052310	33	0
**W01**	**Wang**	15.749760, 100.246513	27	0
**W02**		19.518609, 100.927440	355	300–400
**W03**		18.570830, 100.757160	190	400–500
**W04**		17.665311, 100.298221	69	300–400
**W05**		16.828095, 100.263481	49	0
**Y01**	**Yom**	15.900531, 100.306799	28	0
**Y02**		19.338347, 99.617029	511	5000
**Y03**		18.785228, 99.630360	313	1000
**Y04**		18.267418, 99.456946	224	300–400
**Y05**		17.641443, 99.232070	167	100–200
**YN01**	**Nan**	17.204980, 99.098090	137	100–200
**N01**		19.120022, 100.274112	289	300–400
**N02**		18.115687, 100.114554	155	0
**N03**		17.431125, 99.801378	69	0
**N04**		16.762021, 100.121025	43	0
**N05**		15.940799, 100.254615	34	0
**C01**	**Chao Phraya**	15.550160, 100.114841	21	0
**C02**		15.250528, 100.083250	16	0
**C03**		14.716614, 100.439726	2	0
**C04**		14.304632, 100.566328	2	0
**C05**		13.739310, 100.497611	3	0

### 2.2 PCR inhibition test

According to a prior study's instructions, the removal of PCR inhibitors from water samples was carried out [28]. Internal controls were used to test for inhibition using primers and a probe that target the 16S rRNA of the *Chiropsoides buitendijki*, a marine species that does not live in streams (forward primer: 5′-CCCCAATCGAAATTAAGTTAGCC; reverse primer: 5′-CACAGGTAGAGTGGAGAAATAGAG; probe: 5′-FAM-GTGAAGACGCAGCTTTGTCT-TAMRA-3′). The samples were added with 1.5x10^2^ copies of *C. buitendijki* oligo synthesis (gBlocksTM Gene Fragments, IDT). Inhibition is indicated by a ΔCq or Cq shift of 3 cycles of the internal controls in the water sample from the IPC in the negative controls [29].

### 2.3 Species-specific testing

Extracted DNA was used as a template for qPCR assay together with synthetic fragments. Species-specific primers and probe amplify a 109 bp targeting the mitochondrial cytochrome C oxidase subunit I gene (COI) for the *G. cambodgiensis* were designed and used in the qPCR assays (Supplementary Tab. 1: Accession number of sequences used in the primer design). Sequences of the primers and probe are (Forward primer) GarraCamCOI-F250: 5′-GGGTTTGGAAACTGGCTC-3′, (Reverse primer) GarraCamCOI-R337: 5′-ATAATAGCAGGAATGATGGTGG-3′, and (Probe) GarraCamCOI-P287: FAM 5′-CCCCCGACATGGCATTTC-3′ MGB. The specificity of the primers-probe was first evaluated by in silico analysis using GenBank Primer-BLAST [30]. The qPCR assays were then conducted using mucus DNA to test for the specificity. To minimise potential laboratory contamination, procedures for eDNA extraction from filters and qPCR assays were performed in different rooms. Working areas were bleached and sprayed with 70% EtOH, equipment and materials were also sterilized, and UV treated prior used. Total DNA was extracted from the mucus sample from three individuals *G. cambodgiensis* and one individual of each non-target fish species (co-occurring species) using the Qiagen DNeasy Blood and Tissue Kit (Qiagen, Valencia, CA). qPCR was conducted using species-specific primers which were tested for species specificity with non-target species (co-occurring species) in the same geographic range including *Anabas testudineus, Anguilla bicolor, Barbonymus gonionotus, Channa aurolineatus, Channa micropeltes, Channa striata, Chitala ornate, Cyprinus carpio, Hypsibarbus malcolmi, Labiobarbus spilopluera, Notopterus notopterus, Pangasianodon gigas, Pangasianodon hypophthalmus, Pangasius bocourti, Pangasius larnaudii, Probarbus jullieni,* and *Puntioplites proctozysron.* The qPCR assay was deployed according to Ref. [27]. Six technical replicates of each sample were performed. Negative controls with all qPCR reagents but no template (three replicates) was run in parallel to monitor potential contamination. As positive controls, water samples from fish culture ponds of the species were used in the qPCR experiment.

### 2.4 qPCR of water samples

qPCR of water samples was performed similarly to the mucus DNA tests (see above) with a few minor differences. According to Ref. [31]; there is less bias and credible intervals when at least two samples are taken from each location and analyzed using four to six qPCR repeats. As a result, six qPCR repetitions were done using three replicates of water samples from each sampling site. Cycle threshold (Cq) values of 37.7 or less in at least four of qPCR six replicates were considered positive for *G. cambodgiensis* eDNA detection (Reviews in Refs. [20,32]. The concentration of each sample was calculated based on the synthesized target gene standard curve (Integrated DNA Technologies Pte. Ltd., Singapore) and reported as copies per mL. The limit of detection (LOD) and the limit of quantification (LOQ) were measured using the standard dilution series of synthesized target gene fragments with known copy numbers. A dilution series containing 1.5 × 10-1 to 1.5 × 104 copies per qPCR tube were prepared and used as quantification standards (12 technical replicates). The standard curve for *G. cambodgiensis* (y = −3.5789 × + 42.634, R2 = 0.9912, efficiency = 90.29%) was generated using a 2 μL standard dilution series of each synthesized target. The calculation of LOD and LOQ was done using published R script by Ref. [33]. Random selected positive eDNA detections (of each sampling site) were sent to be sequenced to confirm target species amplification.

### 2.5 Ethics statement

DNA samples of the *G. cambodgiensis* used in this study were extracted from the fish mucus. The *G. cambodgiensis* are not a protected species and no specific permissions were required. After swiping mucus, the fish were released back to where they were found (three individuals). This was approved by the Institute of Animals for Scientific Purposes Development (IAD), University of Phayao under protocol number: 610104004.

## 3 Results

### 3.1 qPCR assay

**Fig. 2:**
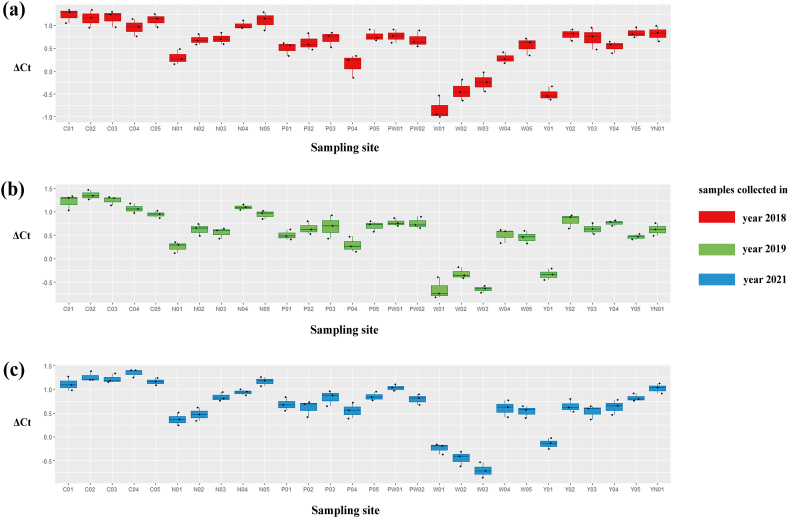
ΔCt values from internal positive controls from PCR inhibition test. Three replicates of samples collected from 28 sites on Chao Phraya River Basin in year (a) 2018, (b) 2019 and (c) 2021. The bold line in the box indicates the median value and upper and lower limits of the box. Whisker plots indicate the first and third quartiles and ±1.5 × interquartile range, respectively. The black dots represent each data point.

Comparing primer sequences of *G. cambodgiensis* with those of closely related and co-occurring species in an in silico analysis revealed mismatches, demonstrating that the primer pair was exceptionally distinctive to the *G. cambodgiensis* (Table 2). Tests of the COI qPCR assay on *G. cambodgiensis* DNA and DNA from non-target fish also showed that the assay is highly specific to *G. cambodgiensis* and demonstrated efficient amplification only from the target species and no amplification from other tested fish. There was no amplification of any of the non-target fish (no amplification after 45 cycles) except for one qPCR replicate (of six) for *Barbonymus gonionotus* and *Notopterus notopterus*, which produced a Cq value of 50.18 and 50.72 (Supplementary F. 1). *G. cambodgiensis* DNA amplified well (Cq values of ∼20–27) for DNA fragment standard and mucus DNA. Limit of detection (LOD), and the limit of quantification (LOQ) was calculated and found to be 25.44 copies/reactions for both values. The average of ΔCq values from the internal controls of all samples were less than 3, which lower than the inhibition criteria (Cq shift of ≥3 cycles was an indication of inhibition) (Fig. 2a, b and 2c). Therefore, PCR inhibition was not likely to occur in all samples.

**Table 2:**
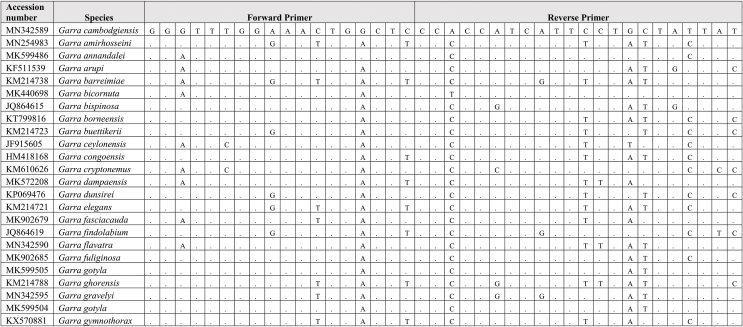

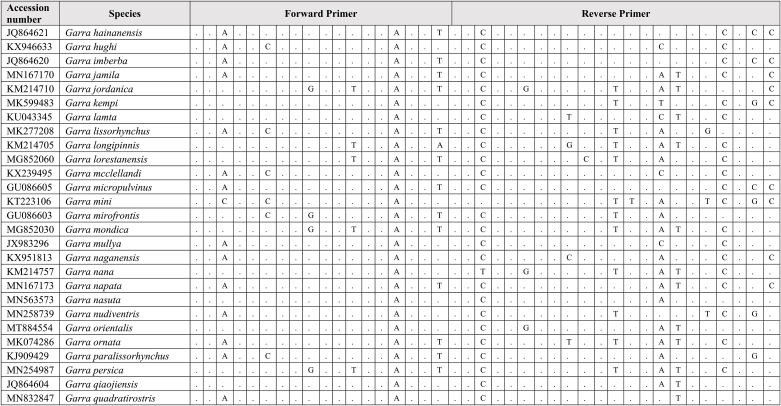

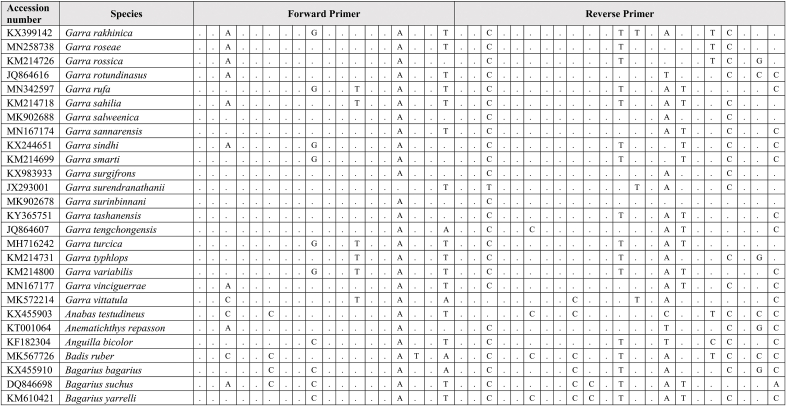

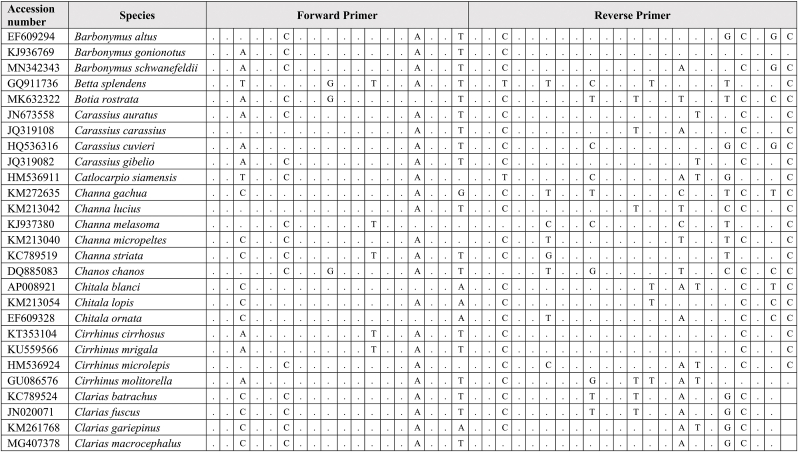

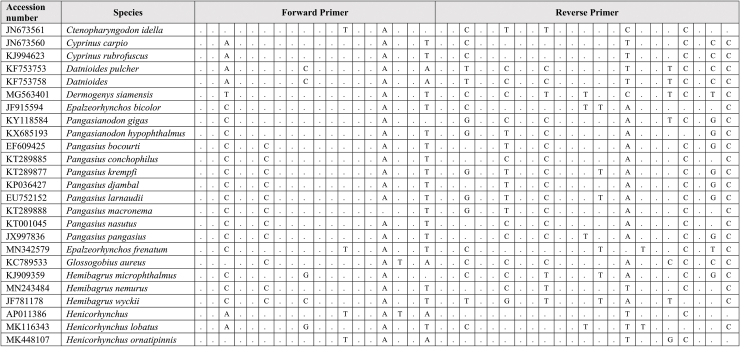

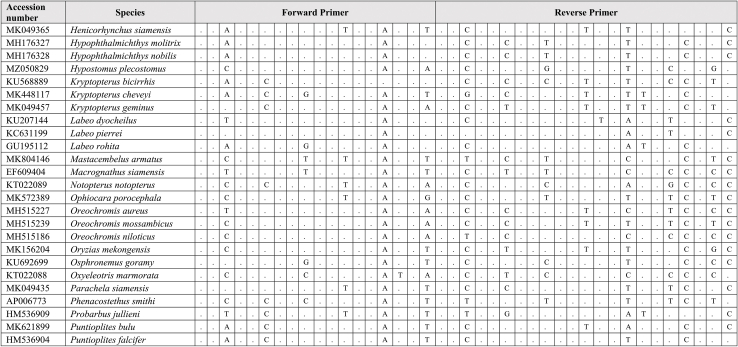

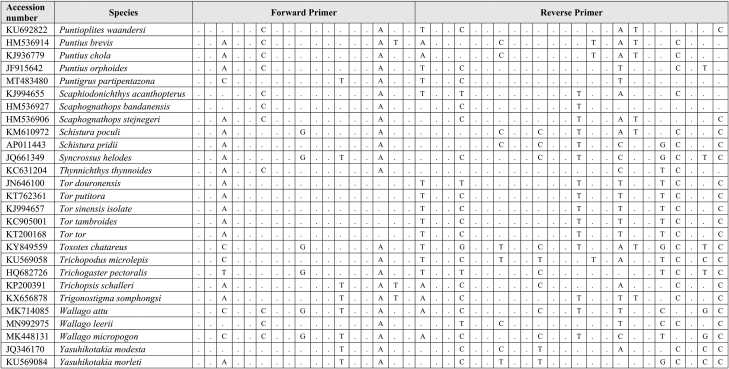
Results of comparing the primer sequences of *G. cambodgiensis* with those of closely related and co-occurring species. Mismatches were shown as A, T, C, G.

### 3.2 eDNA detection in water samples

**Fig. 3:**
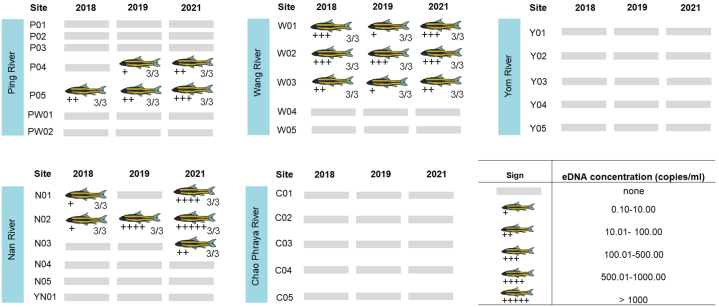
*G. cambodgiensis* eDNA was detected in water samples collected from 28 sites (in five rivers of the Chao Phraya River basin). The eDNA concentration values were represented in “+”, “++”, “+++”, “++++”, and “+++++” as indicated.

*G. cambodgiensis* eDNA was found in one hundred percent of the water samples collected from the pond where *G. cambodgiensis* reside (used as positive controls). The negative control (deionized water) did not produce any DNA amplification. The distribution of *G. cambodgiensis* in the Chao Phraya River basin was determined by qPCR analysis of water samples from five rivers in the basin. For 252 samples (nine filters per one sample, three replicates for each site) corresponding to water samples obtained in 2018, 2019, and 2021, a total of 1512 qPCR reactions were performed. Fewer than half of the obtained water samples contained *G. cambodgiensis* eDNA (78 out of 252 water samples) (Fig. 3 and Table 3). Wang River water samples contained no detectable amounts of eDNA from any *G. cambodgiensis*. *G. cambodgiensis* eDNA was detected only at two sample sites in the Ping Rivers (P04 and P05) and at three sampling sites in the Wang and Nan Rivers (W01, W02, W03, N01, N02, and N03) (Fig. 2). Water samples in 2021 from location N02 (Nan River) contained the greatest DNA content (Fig. 3). *G. cambodgiensis* eDNA was detected in all three replicate water samples at each site where the qPCR experiment was positive.

**Table 3:** eDNA detection of *Garra cambodgiensis* across 28 sites in Thailand.

Site	Sampling year								
	2018			2019			2021		
	eDNA concentration (copie/mL)	Positive samples^a^	eDNA in water samples^b^	eDNA concentration (copie/mL)	Positive samples^a^	eDNA in water samples^b^	eDNA concentration (copie/mL)	Positive samples^a^	eDNA in water samples^b^
**P01**	**-**	0/3	0/18	**-**	0/3	0/18	**-**	0/3	0/18
**P02**	**-**	0/3	0/18	**-**	0/3	0/18	**-**	0/3	0/18
**P03**	**-**	0/3	0/18	**-**	0/3	0/18	**-**	0/3	0/18
**P04**	**-**	0/3	0/18	5.21	18/18	18/18	83.10	3/3	18/18
**P05**	87.47	3/3	18/18	102.99	18/18	18/18	115.52	3/3	18/18
**PW01**	**-**	0/3	0/18	**-**	0/3	0/18	**-**	0/3	0/18
**PW02**	**-**	0/3	0/18	**-**	0/3	0/18	**-**	0/3	0/18
**W01**	204.40	3/3	18/18	4.18	18/18	15/18	234.76	3/3	18/18
**W02**	215.02	3/3	18/18	103.70	18/18	18/18	206.78	3/3	18/18
**W03**	14.31	3/3	18/18	3.34	18/18	16/18	18.41	3/3	18/18
**W04**	**-**	0/3	0/18	**-**	0/3	0/18	**-**	0/3	0/18
**W05**	**-**	0/3	0/18	**-**	0/3	0/18	**-**	0/3	0/18
**Y01**	**-**	0/3	0/18	**-**	0/3	0/18	**-**	0/3	0/18
**Y02**	**-**	0/3	0/18	**-**	0/3	0/18	**-**	0/3	0/18
**Y03**	**-**	0/3	0/18	**-**	0/3	0/18	**-**	0/3	0/18
**Y04**	**-**	0/3	0/18	**-**	0/3	0/18	**-**	0/3	0/18
**Y05**	**-**	0/3	0/18	**-**	0/3	0/18	**-**	0/3	0/18
**YN01**	**-**	0/3	0/18	**-**	0/3	0/18	**-**	0/3	0/18
**N01**	4.58	3/3	16/18	**-**	0/3	0/18	502.02	3/3	18/18
**N02**	6.47	3/3	18/18	506.10	3/3	18/18	3346.50	3/3	18/18
**N03**	**-**	0/3	0/18	**-**	0/3	0/18	19.50	0/3	0/18
**N04**	**-**	0/3	0/18	**-**	0/3	0/18	**-**	0/3	0/18
**N05**	**-**	0/3	0/18	**-**	0/3	0/18	**-**	0/3	0/18
**C01**	**-**	0/3	0/18	**-**	0/3	0/18	**-**	0/3	0/18
**C02**	**-**	0/3	0/18	**-**	0/3	0/18	**-**	0/3	0/18
**C03**	**-**	0/3	0/18	**-**	0/3	0/18	**-**	0/3	0/18
**C04**	–	0/3	0/18	–	0/3	0/18	–	0/3	18/18
**C05**	–	0/3	0/18	–	0/3	0/18	–	0/3	18/18

a Values are the number of positive water samples out of the total number of collected samples (sample replication).

b qPCR replicates positive for *G. cambodgiensis*.

Eight of the 28 water samples collected in 2018 yielded positive qPCR results, with calculated initial copy numbers ranging from 4.58 to 215.02 copies/mL (Table 3). *G. cambodgiensis* eDNA was also found in 6 of 28 water samples collected in 2019, with concentrations ranging from 3 to 506 copies/mL (Table 3). Whereas *G. cambodgiensis* eDNA was found in 8 of 28 water samples taken in 2021, with calculated copy levels ranging from 18.41 copies/mL to 3346.50 copies/mL (Table 3). The positive qPCR detections were Sanger sequenced and checked against the nucleotide BLAST database (NCBI), which proved that they were *G. cambodgiensis* DNA.

Five sites contributing to three rivers (Ping River; P05, Wang River; W01, W02, W03, and Nan River; N02) tested positive for eDNA in each of the three sampling years (Fig. 3). *G. cambodgiensis* eDNA was not detected at location P04 in 2018, but it was detected in 2019 and 2021 (Fig. 2). Interestingly, the identification of eDNA at site N01 was positive in 2018 and 2021, but not in 2019 (Fig. 3). Also *G. cambodgiensis* eDNA was only identified at sampling site N01 in 2021 (Fig. 3). According to the information provided in Table 1, the elevation of all eDNA-positive sites was found to be greater than 140 m. Except for location N03 (69 m), which only shows a positive result in 2021 but not in 2018 or 2019. In addition, all sites with detectable *G. cambodgiensis* eDNA is at least 350 m away from major urban areas (Table 1).

As shown in Fig. 3, the *G. cambodgiensis* eDNA concentration observed in the majority of water samples collected in 2021 with a positive qPCR result (after the COVID-19 pandemic lockdown) increased from 2018 levels (before COVID-19 pandemic). Only at W01 did the concentration of *G. cambodgiensis* eDNA decrease from 215.02 copies/mL in 2018 to 206.78 copies/mL in 2021. Surprisingly, water samples collected at P04 and N03 in 2018 were negative for *G. cambodgiensis* eDNA, but water samples collected in 2021 contained *G. cambodgiensis* eDNA.

## 4 Discussion

The aquaculture and fishing industries were severely impacted by the COVID-19 outbreak. Monitoring, inventory evaluations, and the production and supply system were disturbed [5]. The decisions about conservation and management methods have been impacted negatively [6]. Due to stay-at-home orders prohibiting travel, research, fieldwork, sampling, and tagging programmes have been postponed or cancelled, making it impossible for management to obtain the necessary data. Even during normal times without a pandemic, difficulties in data collection for hard-to-study taxa (e.g., rare, endemic, and endangered) and associated costs are frequently caused by a lack of a complete understanding of organismal distribution and abundance due to the inaccessibility of sampling locations, particularly aquatic environments [34]. Evaluations of fish populations are crucial for environmental monitoring and effective management [35,36]. However, traditional monitoring techniques like as electrofishing, seine, gill net, and trawl are frequently invasive, ecologically harmful, and require a substantial amount of people and money [[37], [38], [39]]. In addition, given the huge spatial scale of aquatic environments such as rivers, it is very hard to conduct accurate evaluations of fish. Moreover, the approaches are influenced by gear type, sample timing, and location in addition to species and size bias [40,41].

Despite this, the assessment of fish distribution in Thailand's major river basins has been a priority for the Thai fisheries department. Unfortunately, it has been hampered by the high expense and labour associated with sampling at broad temporal and spatial scales. Even if a mix of sampling techniques (e.g., trawls and electrofishing) is widely used, incorrect data is still acquired in the majority of cases. Different sampling months and equipment yielded varying results in fish surveys, as an example. *G. cambodgiensis* were captured in three of four sample rounds during a fish survey in Ngim River (Phayao Province, Thailand) in 2019 [14]. In contrast, *G. cambodgiensis* was discovered in every sample month of 2011 in the Nan River (Nan Province, Thailand) using both electrofishing and seine nets [42]. Although the combination of diverse sample methods can provide complementary information regarding the abundance, distribution, and size structure of fish populations, practical constraints and limited monitoring budgets frequently make it challenging to apply on a regional scale. Because monitoring budgets are one of a primary concern for effective conservation management decision-making, rigorous and cost-effective monitoring surveys are required to give precise information on the ranges and abundances of species of interest. Recent environmental DNA sampling has proven to be a highly efficient, trustworthy, and non-destructive tool for monitoring the spatial and temporal distribution of aquatic species (e.g., Refs. [32,[43], [44], [45]]. Previous research indicates that using eDNA in a compromise with tradition methods could yield better detection results or that eDNA can be more sensitive for detecting species than traditional methods such as capture-based surveys (e.g. Refs. [[46], [47], [48], [49], [50]], visual search surveys (e.g. Refs. [48,51], and acoustic surveys [52]. Also, when running costs were considered, eDNA surveying was found to be less expensive than conventional surveying in a number of instances [21,[53], [54], [55]].

eDNA-based survey has also emerged as a valuable tool for generating species distribution models (SDMs) that facilitate ecological inferences at landscape and global scales. SDMs integrate eDNA data with environmental variables to predict species occurrence and understand how climate influences the distribution of fish and other species. Several studies have used eDNA-based approach to investigate species distributions and climate relationships. For instance Ref. [56], applied eDNA metabarcoding to assess the distribution patterns of bony fish communities, revealing the influence of environmental factors, including climate, on species distributions. In addition, eDNA data can yield important ecological inferences at various scales. On the landscape scale, eDNA can help identify suitable habitats, biodiversity hotspots, characterise species assemblages, and inform conservation and management strategies. For instance Ref. [24], used eDNA to map the distribution of newt species, aiding in the identification of critical habitats and guiding conservation efforts. Blackman et al. (2021) also used eDNA metabarcoding to infer fish species distributions and patterns in the Chao Phraya catchment, Thailand. The results from this study were useful for conservation and habitat protection in the study area. At the global scale, eDNA-based data contribute to understanding large-scale biodiversity patterns, identifying areas of high conservation priority, and evaluating the impacts of climate change on species distributions. For example [57], employed eDNA metabarcoding to assess the global distribution of marine fishes, revealing biogeographic patterns and highlighting regions with high species richness and endemism.

Here, effect of the COVID-19 lockdown on fish distribution was mainly investigated. Due to the business collapse in the metropolis caused by the pandemic, a fascinating fact is that many individuals have returned to their hometowns. The increase of inexperienced fishermen appears to induce harmful fishing and a tendency to harvest fish species with a high extinction risk [58]. Herein lies the need for an eDNA-based survey as *G. cambodgiensis* populations are presently in steep decline [14]. Therefore, this study introduces and implements eDNA-based monitoring as a viable alternative to traditional methods for assessing fish distribution. It demonstrates the usefulness of eDNA in revealing the likely dispersal of *G. cambodgiensis* in Thailand prior to and after the COVID-19 lockdown. The findings suggest that the COVID-19 lockdown may have inadvertently benefited the *G. cambodgiensis* by leading to higher concentrations of its eDNA in water samples collected after the lockdown. This indicates the potential for a substantial restocking of the fish population.

While the COVID-19 pandemic has presented numerous challenges to various sectors, including fisheries, it is important to note that there have been some potential positive effects on the restocking of fish populations. Research on the impacts of the COVID-19 outbreak on the environment has now come out. It was discovered that travel restrictions, reduced tourism, and a major drop in social and economic activity had favourable (mainly short-term) effects for animals, the ecosystem, and climate (e.g., Refs. [[59], [60], [61]]. Reduction of energy-related emissions, agriculture-related environmental pressures, usage of non-metallic minerals, and restoration of biodiversity were all positive outcomes of the pandemic (e.g., Refs. [[62], [63], [64], [65], [66]]. Reduced market demand and the closure may boost the likelihood of brood fish survival and replenish fish stocks in some locations [67]. In addition, the decreased human activity during the pandemic has allowed for habitat recovery and restoration. With reduced pollution, sedimentation, and disturbance, aquatic ecosystems have had a chance to rejuvenate, providing more suitable habitats for fish and promoting natural recruitment. Similar to what we discovered here with *G. cambodgiensis* in Thailand, the restrictions and lockdown measures imposed during the pandemic have substantially decreased fishing pressure in many areas. Apparently, the lockdown could be a boon and a significant replenishment of our targeted fish (*G. cambodgiensis*). After the nationwide curfew and partial lockdown in Thailand in 2020, the concentration of *G. cambodgiensis* eDNA increased at all nine sampling sites where the species had been detected prior to the COVID-19 pandemic (2018 and 2019). In addition, at one location (N01) where no *G. cambodgiensis* eDNA was discovered in 2018 and 2019, a positive eDNA result was observed in 2021. However, we cannot conclude with certainty at this time whether the rise in eDNA concentration is a direct result of the increase in fish abundance/biomass; more experiments are required. For instance, a study was conducted in a Japanese river to identify a correlation between eDNA concentrations and real fish density estimations acquired from electrofishing (Yamamoto et al., 2016) or snorkelling survey [28]. In addition, such correlation might be also determined utilising aquaria or mesocosm experiments and experimental ponds [28,[68], [69], [70]].

Also, while qPCR was employed to identify eDNA in this work, it appears that it is not optimal for measuring eDNA. In contrast, a new quantitative PCR technology known as digital PCR (dPCR) may yield more reliable results. Many studies have compared the efficacy of qPCR and dPCR in identifying and quantifying eDNA molecules. Both in mesocosm experiments and in natural environments [71,72], found that dPCR provided more precise eDNA concentration quantification and lower variation results than qPCR. In addition [73], evaluated qPCR and dPCR for the identification eDNA presence-absence of target fish in water samples and discovered that dPCR was more sensitive and precise than qPCR at detecting species with low abundance. Similarly [74], shown that dPCR was superior to qPCR in quantifying eDNA with improved precision, particularly at low eDNA quantities. Overall, these studies suggest that dPCR may provide higher precision and accuracy for detecting and quantifying eDNA compared to qPCR. However, the choice of method may depend on the specific research question and the resources available.

In addition, when environmental parameters such as elevation and distance from urban areas were considered, *G. cambodgiensis* eDNA was found in most samples collected at an elevation greater than 140 m and at least 350 m away from urban areas. Therefore, there is a need to integrate eDNA monitoring with conventional ecological knowledge, such as selecting sampling periods when the target organism is active and/or sampling in locations where the species is likely to be found, etc. Because fish ecological/biological factors such as body size, habitat and diet preference, level of tolerance to water pollution, and ecosystem size may influence eDNA detection in large-scale surveys [[75], [76], [77]].

It is also important to note that the positive effects of the pandemic on fish restocking efforts may vary across regions and depend on various factors, including the severity and duration of lockdown measures, the state of fish populations prior to the pandemic, and the effectiveness of fisheries management practises. Local studies and monitoring programmes are necessary to assess the specific impacts and inform targeted management strategies for fish restocking post-pandemic. While there is potential for positive effects of COVID-19 on the *G. cambodgiensis* population in Thailand, comprehensive scientific studies and long-term monitoring are needed to fully understand the ecological consequences of the pandemic on the species populations and the extent of its influence on restocking efforts. Thus, eDNA-based analysis as a valuable survey tool for monitoring fish populations is promising. The results of this study suggest that eDNA analysis can provide valuable insights into species distribution and abundance, especially in situations where traditional methods are limited, and spatial and long-term studies are required.

## 5 Conclusions

Fish management is frequently hampered by the lack of a comprehensive understanding of organismal distribution, despite the fact that good monitoring programmes are a precondition for the planning of the sustainable use of freshwater resources. Here, eDNA-based monitoring of G*. cambodgiensis* was established and utilised to infer its spread across the largest river basin in Thailand (Chao Phraya). We also illustrate the use of eDNA detection to evaluate the impact of COVID-19 on the spread of species, which may have been influenced by the curfew and lockdowns imposed during the outbreak. The detection of eDNA would be valuable as a supplement for assessing *G. cambodgiensis*as well as other organisms. Without a doubt, appropriate fisheries management methods and/or a conservation plan will benefit from the survey's data.

In addition, the results of this investigation suggest both theoretical contributions and practical managerial implications. This work contributes to theory by demonstrating the efficacy of eDNA-based monitoring as a tool for assessing the dispersal of species. It emphasises the significance of incorporating innovative techniques, such as eDNA analysis, into fisheries management practises, particularly during challenging periods like the COVID-19 pandemic. The necessity of incorporating eDNA-based surveillance into routine fisheries management protocols has implications for management. By combining eDNA analysis with conventional monitoring techniques, administrators can obtain more complete and accurate information on the distribution and abundance of species, allowing them to make informed decisions. In addition, the study emphasises the significance of adapting management strategies to external factors, such as pandemic-related restrictions, and employing innovative tools, such as eDNA analysis, to gain insight into population dynamics and recovery.

## Data accessibility statement

The data that support the findings of this study are available in the Supplementary Material of this article and deposited at Figshare: https://doi.org/10.6084/m9.figshare.21194749.v1.

## Author contribution statement

Maslin Osathanunkul: Conceived and designed the experiments; Performed the experiments; Analyzed and interpreted the data; Contributed reagents, materials, analysis tools or data; Wrote the paper.

Chatmongkon Suwannapoom: Analyzed and interpreted the data; Contributed reagents, materials, analysis tools or data.

## Data availability statement

Data associated with this study has been deposited at Figshare: https://doi.org/10.6084/m9.figshare.21194749.v1.

## Additional information

Supplementary content related to this article has been published online at [URL].

## Declaration of competing interest

The authors declare that they have no known competing financial interests or personal relationships that could have appeared to influence the work reported in this paper.
