# Supplementary material for: eDNA testing reveals surprising findings on fish population dynamics in Thailand
Source: Heliyon. 2023 Jun 10;9(6):e17102. doi: 10.1016/j.heliyon.2023.e17102 (PMC10320040; doi:10.1016/j.heliyon.2023.e17102)
Supplement: Multimedia component 1 [file mmc1.docx]

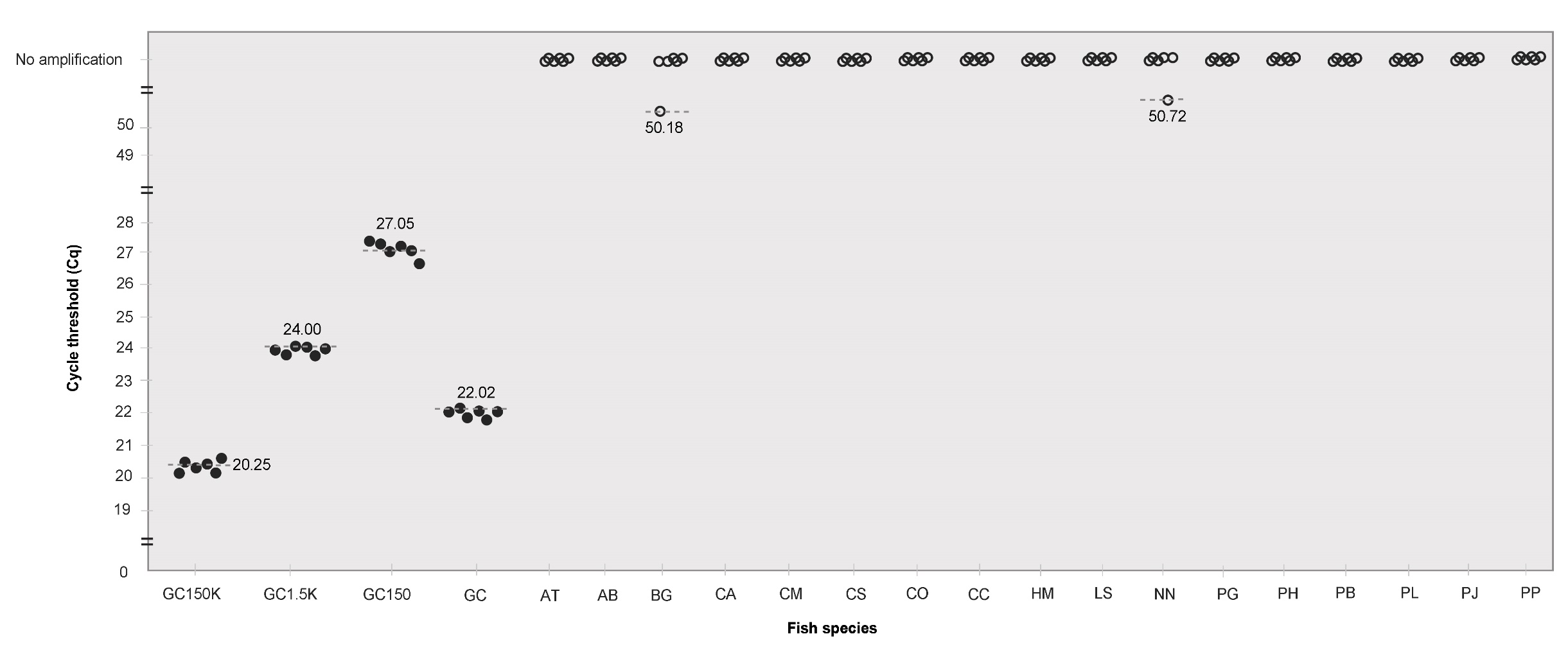


**Supplementary figure 1.** qPCR amplification results for the *G. cambodgiensis* species specific assay. Cycle threshold (Cq) for each reaction (six replicates) for the oligo standards GC150K, GC1.5K, GC150 and for fish mucus DNA) are plotted as circles. Amplification results (positive) for *G. cambodgiensis* DNA labelled as GC150K, GC1.5K, and GC150 represent the 150,000 copy synthesized *G. cambodgiensis* DNA fragment standard, the 1,500 copy fragment standard, and the 150 copy fragment standard, respectively. GC is mucus DNA from *G. cambodgiensis* samples. Fish species tested (non-target fish) are *Anabas testudineus* (AT), *Anguilla bicolor* (AB), *Barbonymus gonionotus* (BG), *Channa aurolineatus* (CA), *Channa micropeltes* (CM), *Channa striata* (CS), *Chitala ornate* (CO), *Cyprinus carpio* (CC), *Hypsibarbus malcolmi* (HM), *Labiobarbus spilopluera* (LS), *Notopterus notopterus* (NN), *Pangasianodon gigas* (PG), *Pangasianodon hypophthalmus* (PH), *Pangasius bocourti* (PB), *Pangasius larnaudii* (PL), *Probarbus jullieni* (PJ), and *Puntioplites proctozysron* (PP).
