# Supplementary material for: eDNA testing reveals surprising findings on fish population dynamics in Thailand
Source: Heliyon. 2023 Jun 10;9(6):e17102. doi: 10.1016/j.heliyon.2023.e17102 (PMC10320040; doi:10.1016/j.heliyon.2023.e17102)
Supplement: Multimedia component 2 [file mmc2.docx]

**Supplementary 1.** Accession number of COI sequences used in primer design

| **Species** | **COI** |  | **Species** | **COI** |
| --- | --- | --- | --- | --- |
| *Garra cambodgiensis* | MN342588 |  | *Kryptopterus geminus* | MK049457 |
| *Garra fasciacauda* | JQ864618 |  | *Labeo dyocheilus* | KU207144 |
| *Anabas testudineus* | KX455903 |  | *Labeo pierrei* | KC631199 |
| *Anematichthys repasson* | KT001064 |  | *Labeo rohita* | GU195112 |
| *Anguilla bicolor* | KF182304 |  | *Mastacembelus armatus* | MK804146 |
| *Badis ruber* | MK567726 |  | *Macrognathus siamensis* | EF609404 |
| *Bagarius bagarius* | KX455910 |  | *Notopterus notopterus* | KT022089 |
| *Bagarius suchus* | DQ846698 |  | *Ophiocara porocephala* | MK572389 |
| *Bagarius yarrelli* | KM610421 |  | *Oreochromis aureus* | MH515227 |
| *Barbonymus altus* | EF609294 |  | *Oreochromis mossambicus* | MH515239 |
| *Barbonymus gonionotus* | KJ936769 |  | *Oreochromis niloticus* | MH515186 |
| *Barbonymus schwanefeldii* | MN342343 |  | *Oryzias mekongensis* | MK156204 |
| *Betta splendens* | GQ911736 |  | *Osphronemus goramy* | KU692699 |
| *Botia rostrata* | MK632322 |  | *Oxyeleotris marmorata* | KT022088 |
| *Carassius auratus* | JN673558 |  | *Pangasianodon gigas* | KY118584 |
| *Carassius carassius* | JQ319108. |  | *Pangasianodon hypophthalmus* | KX685193 |
| *Carassius cuvieri* | HQ536316 |  | *Pangasius bocourti* | EF609425 |
| *Carassius gibelio* | JQ319082 |  | *Pangasius conchophilus* | KT289885 |
| *Catlocarpio siamensis* | HM536911 |  | *Pangasius krempfi* | KT289877 |
| *Channa gachua* | KM272635 |  | *Pangasius djambal* | KP036427 |
| *Channa lucius* | KM213042 |  | *Pangasius larnaudii* | EU752152 |
| *Channa melasoma* | KJ937380 |  | *Pangasius macronema* | KT289888 |
| *Channa micropeltes* | KM213040 |  | *Pangasius nasutus* | KT001045 |
| *Channa striata* | KC789519 |  | *Pangasius pangasius* | JX997836 |
| *Chanos chanos* | DQ885083 |  | *Pangasius sanitwongsei* | KC627282 |
| *Chitala blanci* | AP008921 |  | *Parachela siamensis* | MK049435 |
| *Chitala lopis* | KM213054 |  | *Phenacostethus smithi* | AP006773 |
| *Chitala ornata* | EF609328 |  | *Probarbus jullieni* | HM536909 |
| *Cirrhinus cirrhosus* | KT353104 |  | *Puntioplites bulu* | MK621899 |
| *Cirrhinus mrigala* | KU559566 |  | *Puntioplites falcifer* | HM536904 |
| *Cirrhinus microlepis* | HM536924 |  | *Puntioplites waandersi* | KU692822 |
| *Cirrhinus molitorella* | GU086576 |  | *Puntius brevis* | HM536914 |
| *Clarias batrachus* | KC789524 |  | *Puntius chola* | KJ936779 |
| *Clarias fuscus* | JN020071 |  | *Puntius orphoides* | JF915642 |
| *Clarias gariepinus* | KM261768 |  | *Puntigrus partipentazona* | MT483480 |
| *Clarias macrocephalus* | MG407378 |  | *Scaphiodonichthys acanthopterus* | KJ994655 |
| *Ctenopharyngodon idella* | JN673561 |  | *Scaphognathops bandanensis* | HM536927 |
| *Cyprinus carpio* | JN673560 |  | *Scaphognathops stejnegeri* | HM536906 |
| *Cyprinus rubrofuscus* | KJ994623 |  | *Schistura pridii* | AP011443 |
| *Datnioides pulcher* | KF753753 |  | *Schistura poculi* | KM610972 |
| *Datnioides undecimradiatus* | KF753758 |  | *Syncrossus helodes* | JQ661349 |
| *Dermogenys siamensis* | MG563401 |  | *Thynnichthys thynnoides* | KC631204 |
| *Epalzeorhynchos bicolor* | JF915594 |  | *Tor douronensis* | JN646100 |
| *Epalzeorhynchos frenatum* | MN342579 |  | *Tor putitora* | KT762361 |
| *Glossogobius aureus* | KC789533 |  | *Tor sinensis* | KJ994657 |
| *Hemibagrus microphthalmus* | KJ909359 |  | *Tor tambroides* | KC905001 |
| *Hemibagrus nemurus* | MN243484 |  | *Tor tor* | KT200168 |
| *Hemibagrus wyckii* | JF781178 |  | *Toxotes chatareus* | KY849559 |
| *Henicorhynchus lineatus* | AP011386 |  | *Trichopodus microlepis* | KU569058 |
| *Henicorhynchus lobatus* | MK116343 |  | *Trichogaster pectoralis* | HQ682726 |
| *Henicorhynchus ornatipinnis* | MK448107 |  | *Trichopsis schalleri* | KP200391 |
| *Henicorhynchus siamensis* | MK049365 |  | *Trigonostigma somphongsi* | KX656878 |
| *Hypophthalmichthys molitrix* | MH176327 |  | *Wallago attu* | MK714085 |
| *Hypophthalmichthys nobilis* | MH176328 |  | *Wallago leerii* | MN992975 |
| *Hypostomus plecostomus* | MZ050829 |  | *Wallago micropogon* | MK448131 |
| *Kryptopterus bicirrhis* | KU568889 |  | *Yasuhikotakia modesta* | JQ346170 |
| *Kryptopterus cheveyi* | MK448117 |  | *Yasuhikotakia morleti* | KU569084 |
